# Trends in the Molecular Pathogenesis and Clinical Therapeutics of Common Neurodegenerative Disorders

**DOI:** 10.3390/ijms10062510

**Published:** 2009-06-03

**Authors:** Yahya E. Choonara, Viness Pillay, Lisa C. du Toit, Girish Modi, Dinesh Naidoo, Valence M.K. Ndesendo, Sibongile R. Sibambo

**Affiliations:** 1 University of the Witwatersrand, Department of Pharmacy and Pharmacology, 7 York Road, Parktown, 2193, Johannesburg, South Africa; 2 University of the Witwatersrand, Department of Neurology, 7 York Road, Parktown, 2193, Johannesburg, South Africa; 3 University of the Witwatersrand, Department of Neurosurgery, 7 York Road, Parktown, 2193, Johannesburg, South Africa

**Keywords:** Parkinson’s disease, Alzheimer’s disease, Amyotrophic Lateral Sclerosis, Huntington’s disease, neuropathology, amyloid-β protein, Tau, Huntingtin, α-Synuclein, neurotherapeutics, drug delivery

## Abstract

The term neurodegenerative disorders, encompasses a variety of underlying conditions, sporadic and/or familial and are characterized by the persistent loss of neuronal subtypes. These disorders can disrupt molecular pathways, synapses, neuronal subpopulations and local circuits in specific brain regions, as well as higher-order neural networks. Abnormal network activities may result in a vicious cycle, further impairing the integrity and functions of neurons and synapses, for example, through aberrant excitation or inhibition. The most common neurodegenerative disorders are Alzheimer’s disease, Parkinson’s disease, Amyotrophic Lateral Sclerosis and Huntington’s disease. The molecular features of these disorders have been extensively researched and various unique neurotherapeutic interventions have been developed. However, there is an enormous coercion to integrate the existing knowledge in order to intensify the reliability with which neurodegenerative disorders can be diagnosed and treated. The objective of this review article is therefore to assimilate these disorders’ in terms of their neuropathology, neurogenetics, etiology, trends in pharmacological treatment, clinical management, and the use of innovative neurotherapeutic interventions.

## 1. Introduction

Disorders that are associated with atrophy of the central nervous system (CNS) structures are termed neurodegenerative disorders (NDs) with the most common of these disorders being Alzheimer’s disease (AD), Parkinson’s disease (PD), Amyotrophic Lateral Sclerosis (ALS) and Huntington’s disease (HD). The prevalence of NDs is increasing rapidly despite extensive research in the field [1]. The disorders are both sporadic and familial and affect distinct zones of the brain, causing motor and/or cognitive difficulties such as aphasia, apraxia and agnosia that consequently disrupt the personality, intellect, social and occupational function of patients [2–6].

**Figure 1.**
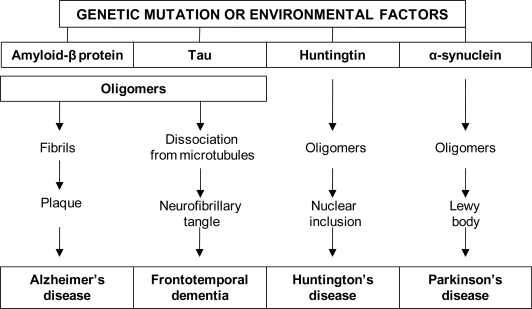
Schematic diagram outlining the pathogenesis of common neurodegenerative diseases (Adapted from Yuan and Yanker, [16]).

Due to an overlap in the neuropathological symptoms of common NDs a fundamental challenge is the identification of genetic patterns and symptoms that reveal the type of disorder whilst the patient is still alive. These uncertainties often lead to misdiagnosis and subsequent erroneous therapy. The rapidly growing insights into the genetic and molecular mechanisms underlying NDs need to be detailed to ensure superior diagnostic strategies and to aid in exchanging knowledge between scientists and health professionals. Evaluation of prevalent genetic mutations facilitates the identification of genetic markers that may be associated with NDs. Research conducted on various animal models and human epidemiological studies demonstrated clear evidence verifying that genetic and environmental factors may lead to an increased risk of neurodegeneration [7–9]. Some NDs are characterized by neuronal and glial accumulations (since inclusions are mainly deposited in astroglia) [10–14]. Tauopathies associated with NDs have several commonalities at the molecular level and many are characterized by age-dependent abnormal accumulation of misfolded proteins that aggregate into fibrillar deposits with amyloid. The extent of neurodegeneration depends on the area and magnitude of protein aggregation. Aggregation of abnormal proteins both inside and outside cells is a prominent feature of common disorders such as AD and PD [15]. Similarly, synucleinopathies, e.g. PD, are characterized by aggregation of α-synuclein and, occasionally, gene mutations (Figure 1).

Although NDs are associated with progressive neuronal loss, a few neurological impairments may reflect network dysfunction rather than a loss of neurons [17–19]. Patients in particular with AD or other NDs display remarkable fluctuations in neurological function even during the same day. These fluctuations most likely reflect variations in neural network activity such as chronic intoxication by abnormal proteins that the brain is temporarily able to overcome [20,21]. Thus far, abnormal protein aggregation have shown to trigger cycles of aberrant neuronal activity and compensatory alterations in neurotransmitter receptors with related signaling pathways that lead to synaptic deficits, disintegration of neural networks, and ultimately failure of neurological function [21]. *In vivo* studies in animal models have shown that few pathogenic cascades can be prevented or reversed by removing abnormal proteins or pharmacologically modulating neuronal activity without precipitating effects on neuronal number [22]. Enhancing neuronal plasticity may help functional neural circuits to compensate for nonfunctional circuits and improve the overall network performance and neurological function [20,23,24] Furthermore, apoptosis involvement in NDs has been detected in autopsies of human brain tissue as well as in *in vivo* animal models [25,26]. Understanding the central role of cascades during apoptosis has led to the development of several inhibitory neuroactive drugs. Post-mortem investigations have enlightened researchers on the microglia-produced inflammatory and neurotoxic factors such as cytokines and tumor necrosis factor-α (TNFα) that have free radicals which are deleterious to neurons [27].

Developing new diagnostic and treatment modalities for NDs is an active area of research that attempts to evaluate current therapeutic interventions towards restoring neurochemical balances in the brain or slowing the progression of NDs [28–31]. The few neuroactive drugs that are currently approved by the US Food and Drug Administration (FDA) have demonstrated only modest effects in modifying the symptoms of NDs for relatively short periods of time in subsets of patients, and none has shown an effect on halting the progression of NDs. Pharmaceutical researchers are confronting past failures, and therefore tissue transplantation and stem cell research are currently a major area of investigation. Mobilizing endogenous neuronal stem cell populations to repair damaged tissue and potentially stimulate reformation of damaged synapses is being explored [32–34]. A discerning fact on neurotherapeutics is the constraint of the Blood-Brain Barrier (BBB) and the drug release kinetics that cause increased side-effects. The vascular endothelial cells, choroid plexus and the arachnoid membrane act together to form a barrier between the blood and cerebrospinal fluid to efficiently prevent neuroactive agents from entering the brain. More in depth exploration of the BBB would aid in the design of techniques to manipulate the BBB and transport neuroactive agents into the brain.

This review paper attempts to identify and assimilate the key molecular features of common NDs and the associated mutated protein inclusions in the degenerating cells and the surrounding viable neurons. AD, PD, ALS and HD are extremely complex and common NDs and have been selected for review in this paper. Their etiology is frequently unknown, however severe genetic mutations that lead to misfolding and aggregation in extracellular protein depositions coupled with neuronal network dysfunction have been observed in NDs. Currently, there are no completely effective neurotherapeutic interventions that have been developed. Research findings that implicate the commonalities in protein aggregations could insinuate that a few NDs may share a common or overlapping neuropathogenic mechanism(s) which could be targeted by synchronized neurotherapeutic strategies. Therefore, in addition this review paper provides a concise incursion into the neurotherapeutic modalities directed to CNS drug delivery that may be useful in overcoming many of the therapeutic challenges of NDs. To meet this end, the neuropathology and genetics of common NDs, the current neurotherapeutic trends of NDs, the possible novel biomarkers that can be used to identify the debilitating symptoms of neurodegeneration, and recent directions in research and clinical trials are also discussed.

## 2. General Underlying Mechanisms Resulting in Neurodegeneration

### 2.1. Network dysfunction

Due to the fact that the progressive neurological decline observed in NDs is associated with neuronal loss, the peak performance of patients at any disease stage may be capped by the loss of neurons. However, the extent to which neurological deficits in NDs relate directly to neuronal loss is controversial [17,18,22–24]. Furthermore, processes that do not involve significant neuronal loss may cause similar functional impairments. Thus, it is unlikely that changes in neuronal number may account for the rapid and reversible fluctuations in neurological function. Such fluctuations probably reflect complex adjustments in molecules, signaling cascades, synaptic modifications, neuronal activities and network interactions. It has been established that transgenic mouse models expressing abnormal proteins develop distinct disease-related neurological impairments [21]. In transgenic mice, the elimination of abnormal proteins may reverse neurological deficits without changing neuronal number [35]. Thus, neurological impairments that are associated with NDs may be caused by neuronal dysfunction rather than neuronal loss. This concept is supported by the fact that effective neural plasticity allows the brain to cope even with striking neuronal losses [36].

### 2.2. Synaptic dysfunction leading to network failure

Neural plasticity is highly adaptive during healthy and disease states. This enables individuals to interact effectively with their environment and to cope with neural injuries. A major component of neural plasticity is found in synapses, which are actively strengthened and weakened by complex processes to form the dynamic neural circuits and higher-order networks that store memories, give rise to thoughts and create the awareness of an individual. Notably, abnormal proteins that are suspected of causing NDs impair the integrity or function of pre-synaptic terminals and post-synaptic specializations [37]. Numerous mechanisms may be involved, including excitotoxicity [38], inflammation [39], oxidative stress [40] and other processes [41–43]. In AD synaptic loss exceeds neuronal loss, and depletion of synapses and synaptic proteins correlates with cognitive decline than does the abundance of plaques or tangles [43]. Chronic alterations in synaptic plasticity and neurotransmission may affect activity-dependent signaling and gene expression, resulting in the disintegration of neural networks which ultimately leads to the failure of neural function. Conversely, environmental stimulation has shown to increase synaptic plasticity that may delay and decrease pathological alterations [44]. Furthermore, diverse neural injuries elicit effective plasticity responses with the protective apo-E2 and apo-E3 isoforms, but not with apo-E4 which increases the risk and accelerates the onset of AD and PD [45].

### 2.3. Survival of neurons

There are numerous factors that may influence the survival of neurons at different stages of NDs. These include the functional state of neurons in affected areas [46,47], the ability of people to use specific learning strategies to overcome deficits [48,49], genetic factors such as apo-E isoforms [45] and co-morbidities such as vascular disease [50]. In addition, alternative neural networks may compensate for the lost neurons [47,48]. However, such compensation of neuronal dysfunction does not directly influence the survival of neurons, but rather the phenotype. The prototypic elements of the Fibroblast Growth Factor (FGF) family, FGF1 and FGF2, have been implicated in various physiological and pathological processes. FGF1 and FGF2 have been ubiquitously expressed however are not efficiently secreted. Gene knockouts in the mice model have previously demonstrated a role for FGF2 in brain development. Studies of mice lacking individual FGFs revealed a variety of phenotypes that ranged from early embryonic lethality to mild defects These findings most likely reflect the redundancy of the FGF family of ligands or their uniqueness of expression in specific tissues [51]. The relatively mild phenotypic defects associated with FGF2 deletion led to the hypothesis that the continued expression of other FGFs partially compensated for the absence of FGF2 in the mice. Miller and coworkers, [51], reported the generation of mice lacking FGF1 and their use, in combination with a previously described FGF2 null mouse, to produce mice that lack both FGF1 and FGF2. FGF1-FGF2 double-knockout mice were found to be viable and fertile and did not display any gross phenotypic defects. In the double-knockout mice they observed defects that were similar in extent to those previously described for the FGF2 null mice. In addition, differences in the organization of neurons of the frontal motor cortex were observed and no abnormalities were found in mice lacking only FGF1. Their results suggested that the relatively mild defects in FGF2 knockout animals were not a consequence of compensation by FGF1 and suggested highly restricted roles for both factors under normal neuronal developmental and physiological conditions [52–55].

Most modulators represent potential targets for therapeutic intervention. Studies have shown that in the absence of disease, neural systems are characterized by extensive degeneracy i.e. the ability of structurally different elements to perform the same function or yield the same output [56–58]. This degeneracy may serve to explain the fact that neurons in the substantia nigra may become nonfunctional before they are missed at the clinical level and cause NDs to progress at a gradual pace. The complexity of compensatory mechanisms has been demonstrated by the multi-level adjustments that can occur in the cortical–basal-ganglia–thalamocortical network at different stages of PD and in related animal models [59]. Notably, progressive failure of these compensatory mechanisms may be the contributing factors to the prominent daily fluctuations in the effectiveness of levodopa which is often referred to as “the on–off phenomenon” in advanced PD treatment. Therefore, tremendous benefits may be reaped from neurotherapeutic interventions that maximize the patient’s opportunity for optimal performance by shifting the focus from neurons that are lost to those that survive [60]. In addition, several studies reporting the survival, integration and function of stem cell grafts in animal models of HD have been reported [61–63]. The majority of these studies have been based on transplantation of fetal neural progenitors, expanded as neurospheres and differentiated *in vitro* prior to transplantation [64].

## 3. Overview of Alzheimer’s Disease

Alzheimer’s disease (AD) is recognized as the most common form of dementia affecting an estimate of 24 million people worldwide, 40% of them living in less developed countries [65]. The prevalence of dementia in Western Europe is about 4.5 million, and its total annual costs amount to 55 billion Euros [65–67]. As a consequence of rapid demographic ageing, AD has become one of the most severe progressive socio-economical and medical burdens facing countries in the next century. AD can be classified into early onset (< 65 years) and late onset (> 65 years) forms [68]. Sporadic AD is the most common form of both early- and late-onset AD. It is considered to be a result of complex genetic and environmental risk factors. AD is characterized by a progressive decline in cognitive function, loss of memory, and other neuro-behavioural symptoms. The progressive neurodegeneration occurs in multiple areas of the brain, including the nuclei basalis, hippocampus, amygdale, and entorhinal cortex. Although the etiology of AD is unknown and the pathophysiological processes that underlie the steady progression of the clinical course are not clearly understood, it is apparent, that mechanisms of cell-to-cell communication are disrupted. Genetic factors may also play a substantial role in the etiology of adult-onset AD [69].

There is no laboratory test for the diagnosis of AD. The diagnosis of AD is primarily based on clinical and neuropsychological observations with the simultaneous exclusion of other possible causes for dementia. The most commonly used clinical criteria were outlined previously by the National Institute of Neurological and Communicative Disorders and Stroke and Alzheimer’s Disease and Related Disorders Association (NINCDS-ADRDA) [70]. According to NINCDS-ADRDA, the diagnosis of AD may be confirmed by neuropathological examination at either biopsy or autopsy using biological markers (neuro-imaging). The diagnosis made by the NINCDS-ADRDA has high accuracy (80 – 90%) according to data obtained from patients with severe AD and repeated examinations [71,72]. However, the accuracy is acknowledged to be lower when diagnosing patients with early stages of AD or with mild cognitive impairment (MCI), and in the absence of repeated examinations [73].

The clinical diagnosis of AD is currently based on progressive memory impairment and decline in at least one other cognitive domain, and by excluding other diseases such as frontotemporal dementia, dementia with Lewy-bodies (DLB), stroke, brain tumor, normal pressure hydrocephalus or depression, that might also present with dementia [74,75]. The clinical diagnostic accuracy for AD depends on the stage of disease and can exceed 90% [76]. Diagnostic criteria for AD have been proposed within both the Diagnostic and Statistical Manual (DSM) [77] and International Classification of Diseases (ICD) classification systems [78]. However, the criteria followed in most research studies are those proposed by the NINCDS-ARDA for defining probable AD [70]. A variable period of prodromal decline in cognition of up to five years usually precedes the formal diagnosis of AD. This stage, known as Minimal Cognitive Impairment (MCI), is characterized by a relatively isolated impairment in long term memory and may also be accompanied by impairments of working memory. These deficits presumably relate to damage to the medial temporal lobe and/or specific prefrontal–temporal lobe circuits. Approximately 40 – 60% of subjects with MCI will subsequently progress to meet criteria for AD over a 3-4-year period [79–81].

AD is a pathologically complex and etiologically a multi-factorial disease. There are a few causative genes which have been linked to the relatively small proportion of patients with early-onset familiar AD. In addition, numerous other genetic risk factors predisposing for AD have been identified [82]. However, it has become evident that genes alone cannot explain the late-onset sporadic AD. Numerous environmental factors also contribute significantly to the development of AD. In the last decade significant progress has been made in elucidating the resulting changes of single-gene defects in familial forms of AD [83]. For instance it has been established that a genetic defect that causes a severe immune deficiency in humans may also produce balance disorders [84]. Several highly penetrant genes have been cloned for rare, autosomal-dominant, early onset forms of AD. The major gene defects include mutations in the amyloid precursor protein and presenilins that lead to increased amyloid deposit and AD [83]. Gene defects are responsible for approximately 1% of all AD cases and currently numerous efforts are being directed to searching for mutations in identified genes with the hope that this may provide further insight into the more common sporadic cases of AD [83].

### 3.1. The neuropathology of Alzheimer’s disease

Realization of accurate pathophysiology is vital for the development of neuroactive agents that are able to cease the β-amyloid (Aβ) peptide aggregation and avert the progression of AD. The neuropathological features of AD is the fibrillar aggregates of Aβ peptides in senile plaques and blood vessel walls, the formation of neurofibrillary tangles (NFTs), and the loss of neurons, which lead to the compromised functioning of the neurotransmitter system. While Aβ peptides are the primary components of AD plaques, there have been a few discrepancies on whether the Aβ aggregation is the only cause of AD neurotoxicity [85]. On one hand, there is considerable evidence that fibrillar aggregation of free, non-polymerized Aβ and amyloid deposition are associated with AD neurotoxicity. In addition, there have also been propositions that other neuropathological abnormalities such as gliosis, chronic inflammatory reactions, excitotoxic damage and oxidative stress appear to be also involved in the neurodegenerative processes occurring in the AD brain [76].

#### 3.1.1. The amyloid hypothesis

The amyloid hypothesis as the primary trigger of AD is supported by the fact that all known mutations causing familiar AD target Amyloid Precursor Protein (APP) processing and that plaque formation has also been estimated to be a relatively early event in AD brain [86]. The Aβ plaques and NFTs are intracellular aggregates of hyperphosphorylated microtubular tau proteins appearing as paired and helical filaments [13]. Non-polymerized Aβ peptides insert between lipid bi-layer membranes and generate non-selective cation channels that may trigger cellular death. In addition, ubiquitin, a protein that generally latches onto mutated proteins tagging them for proteolysis, is found in association with NFTs. Consequently the ubiquitinated NFTs are recognized for degradation by proteosomes in the brain [86]. The histopathological signs at onset are Aβ plaques which progress to the development of NFTs, neuronal reduction and subsequent brain atrophy [85–87]. These changes are particularly observed in the hippocampus, substantia innominata, locus cerulueus as well as the tempoparietal and frontal cortices of the brain. Further accrued data implicate the neuronal lysosomal system as an additional degradative pathway. Mutations of presenilin 1 and 2 have amyloid-independent effects on the lysosomal system which augment the Aβ plaque [88–91]. In addition to brain atrophy and widened sulci, the neurochemical signs are the paucity of neurotransmitters including choline acetyltransferase, acetylcholine, noradrenalin, serotonin and somatostatin [92]. Changes in neurotransmitter concentrations lead to behavioural changes such as wandering, agitation, depression, aggression and delusions. Neuroactive agents capable of impeding the aggregation may be potentially useful in preventing or diminishing the toxicity of Aβ peptides. In this regard, recent studies have aimed at interfering with amyloid aggregation as a possible route for prevention or treatment of AD [13,86].

#### 3.1.2. The tau hypothesis

Neurofibrillary changes of abnormally hyperphosphorylated tau are the key lesion in AD and a number of other tauopathies [93]. The phosphoprotein, tau that is synthesized by neurons is also present in astrocytes and oligodendrocytes within the brain, and in peripheral tissues [94,95]. Tau is coded by a gene located on chromosome 17 and alternatively spliced into 6 isoforms ranging from 352 – 441 amino acids in length [96, 97]. Tau is the major microtubule-associated protein that promotes the assembly and stability of microtubules, which play vital structural and functional roles in neurons. Healthy neurons are internally supported in part by these microtubules that facilitate the transport of nutrients and other cellular components from the cell body to the ends of the axon and back of neurons. Tau, which normally has phosphate molecules attached, binds to the microtubules and stabilizes them. In AD, an abnormally high number of additional phosphate molecules become attached to tau. As a result, tau disengages from the microtubules and aggregates forming tangles. The microtubules disintegrate and the transport system of the neuron collapses resulting initially in malfunctions in communication between neurons and later in neuronal cell death. Tau phosphorylation has been suggested to play a key role in AD pathogenesis since it has been shown to correlate with the severity of dementia. Braak and Braak, [98,99], proposed a model of pathological evolution of AD. According to the model, NFTs appear in the entorhinal cortex during the preclinical phase, spreading to the hippocampus in the middle phase and, finally, progressed to the neocortex during the late stages of AD [99]. Although scientists are exploring further, currently the etiology of AD still remains a challenge [100–104].

However, the present focus for AD includes endogenous molecules that are associated with Aβ peptide production such as the APP, presenilin 1 and 2 genes located on chromosome 21, 14 and 1 respectively [105,106]. Chemically altered tau proteins instead, aggregate to form disabling tangles that injure the nerve cells. Moreover, once microtubules disintegrate the transport system of the neurons collapse leading to communication malfunctions between neurons and later also in brain cells. The neuron disintegration along with tau-based traffic inhibition may represent the early stages of AD [106].

#### 3.1.3. The role of inflammation

Inflammatory reactions in the CNS are mainly mediated by brain-specific glial cells, notably by microglia and astrocytes, and to a lesser degree by neurons. Under physiological conditions, glial cells support and protect neurons and their functions, and have numerous critical roles in the homeostasis and activity of the brain [107]. In AD, inflammation has recently been confirmed as an essential feature, particularly in association with neuritic plaques. The inflammatory reaction is characterized by activation of glial cells, gliosis and the appearance of inflammatory proteins such as complement factors, acute phase proteins, and pro-inflammatory cytokines [108]. Evidence from autopsy-based studies suggests the involvement of microglial activation in the pathogenesis of neurodegeneration by the secretion of a variety of pro-inflammatory and neurotoxic factors. These macrophage-like cells were found to co-localize with neurotic plaques in the brain and are believed to induce as well as exacerbate neurodegeneration [109]. This phenomenon was also supported by clinical trials in which non-steroidal anti-inflammatory drugs were demonstrated to reduce the risk of developing AD [110].

A **s**tudy revealed that pre-treatment of primary cultures of microglia generated from rapid-autopsy brain tissue with indomethacin and anti-Aβ antibody resulted in the efficient opsonization and subsequent degradation of Aβ peptides without the concomitant up-regulation of cytokines that are typically associated with the activated state [111]. In addition, Rogers and Lahiri, [112] have identified inflammation cytokines and biological metal such as copper, zinc and iron as significant factors that augment the onset of sporadic late onset forms of AD.

**Figure 2.**
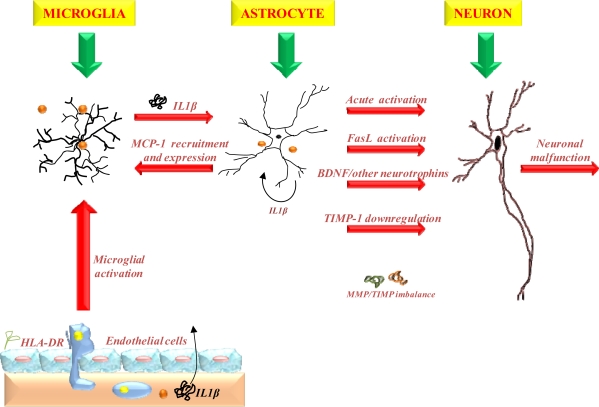
The role of glial cells in central nervous system inflammation and neurodegeneration. BDNF = brain-derived neurotrophic factor, MP = macrophages, MMP = membrane metalloproteinase, TIMP = tissue inhibitors of metalloproteinase (Adapted from: Ghorpade *et al.* [113]).

Ghopade *et al.* [113] are extensively investigating the proposed activation of glia (astrocytes and microglia) by pro-inflammatory cytokines such as IL-1β leading to changes in the immune and neural functions of astrocytes during inflammation ultimately contributing to NDs (Figure 2). The acute inflammatory response of glia includes enhanced production of tissue inhibitor of metalloproteinase (TIMP-1) in the tissue microenvironment that serves as a repair response early in injury. In addition, expression of Fas ligand (a type II transmembrane protein that belongs to the TNF family) and down-regulation of other trophic factors such as brain derived neurotrophic factor (BDNF) during acute immune activation of glial cells may also contribute to neurotoxicity. However, under sustained inflammatory conditions, such as those observed in chronic NDs, the response of glia may be significantly different (i.e. decreased production of TIMP-1). Dhar *et al.* [114] previously implicated the importance of astrocyte production of tissue inhibitor of TIMP-1 in CNS homeostasis and inflammatory diseases. The contribution of transforming growth factor (TGF)-signaling in astrocyte-MMP/TIMP-1-astrocyte regulation was investigated. Co-stimulation of astrocytes with IL-1β and TGF mimicked the TIMP-1 down-regulation observed with IL-1β chronic activation. The study proposed that one of the mechanisms involved in TIMP-1 down-regulation may be through TGF-signaling in chronic immune activation, thus demonstrating a novel extracellular regulatory loop in astrocyte-TIMP-1 regulation [114].

#### 3.1.4. The role of free radicals

Oxidative damage of biological systems occurs when the production of oxygen radicals and ROS exceeds the cell’s antioxidant capacity, leading to oxidative imbalance in a system [115,116]. The concept of oxidative stress in AD was originally derived from the free radical theory of aging. Recently, numerous studies have been published reporting a variety of results on the role of oxidative stress in AD [117]. There is evidence of increased oxidation of DNA, RNA, protein, lipids and carbohydrates in the AD brain [118]. Thus, the role of free radicals in the pathogenesis of AD has become an important area of research over the past two decades. Insufficiency in the functioning of mitochondrial cytochrome oxidase, altered iron homeostasis and unbalanced high superoxide dismutase activity, have been identified as primary causes of oxidative damage stress in NDs. In addition, numerous biochemical conditions have been found to produce free-radical malfunctioning by superoxide dismutase. These include mitochondrial cytochrome oxidase insufficiency, unbalanced high activity, and an alteration in iron homeostasis, potentially resulting in the production of more peroxide. *In vitro* studies have shown a role played by oxygen-free radicals in promoting amyloid aggregation. The presence of Aβ plaques induces the susceptibility of neuronal cultures to oxidative toxicity between collaboration of Aβ and oxidative reactive species tend to augment the neuronal damage in AD.

#### 3.1.5. Excitotoxicity

Excitotoxicity is also considered to play an important role in the pathogenesis of AD. It is triggered by excessive stimulation of glutamate receptors (e.g. N-methyl-D-aspartate also known as NMDA) due to either increased release or decreased uptake of excitatory amino acids, mostly glutamate [119]. As a result, perturbations occur in cellular ion homeostasis (Ca^2+^, K^+^, Na^+^) and metabolic activities. The most crucial disturbance is considered to be the overload in the intracellular level of Ca^2+^ that may originate from the extracellular space and/or ER, resulting in diverse neurotoxic effects due to calcium dysregulation and alterations in Ca^2+^-signaling pathways [120,121]. Calcium dysregulation may also be caused by ER stress, which is characterized by inhibition of protein glycosylation and an increase in unfolded proteins in the ER [122,123]. Under pathological conditions, an increase in intracellular calcium results in overload of Ca^2+^ in mitochondria, causing mitochondrial dysfunction manifested by increased production of ROS, decreased energy metabolism, release in cytochrome c, and eventually apoptosis [124]. In brain, calcium dysregulation is considered to be capable of eliciting increased Aβ formation and tau phosphorylation [125,126]. It must be remembered that calcium dysregulation, ER stress, mitochondrial dysfunction, and defective energy metabolism may also be initiated by other factors that are unrelated to excitotoxicity.

#### 3.1.6. Other risk factors

Age and family history are significant risk factors. However environmental factors, previous head injury and Down’s syndrome cannot be ruled out. Other risk factors include polymorphism in genes such as α-microtubulin, apo-E, low density lipoprotein receptor-related protein and very low density lipoprotein receptors. While mutations of the PS-1, PS-2,11 and APP genes (resulting in increased production and elevated plasma levels of Aβ protein) on chromosomes 1, 14, and 21, respectively, have been associated with the rare form of familial AD, the only consistent marker for the late-onset non-familial form of AD is the apo-E allele on chromosome 19. The apo-E gene is present in three alleles, ɛ2, ɛ3, and ɛ4. The ɛ4 allele and α2 macroglobulin polymorphism are major risk factors for causing AD. This refers specifically to the ɛ4 allele, though it is absent in approximately 30 – 40% of patients with AD and present in approximately 30% of healthy subjects. Conversely, the ɛ3 allele is believed to represent no increased or decreased risk, while the ɛ2 allele may confer some protection [64,127,128]. Increasing evidence also suggests that apoptosis is the major type of cell death involved in NDs although inflammation is also involved in the pathogenesis [129–131]. In the CNS, potential inducers of apoptosis include neurotrophic factors, changes in potassium concentration, calcium dysregulation, modulators of protein phosphorylation, DNA damage, neurotransmitters (glutamate), peptides and proteins (Aβ), oxidative stress, nitric oxide, lipids (i.e. retinoic acid), irradiation, as well as certain neurotoxins such as ethanol [132].

### 3.2. Clinical and therapeutic findings on Alzheimer’s disease

The present focus for AD treatment includes endogenous molecules that are associated with Aβ production and clearance such as the β- and γ-secretases, insulin-degrading enzymes namely, neprilysin, and immunotherapeutic-based approaches. Recent evidence from preclinical transgenic mice models proposed that deposited Aβ could be removed by modulating particular endogenous inflammatory processes. Chang *et al.* [133] revealed that the structure-based design of memapsin 2 (β-secretase) inhibitors linked to a carrier peptide inhibits the production of Aβ. Researchers used an intra-peritoneal formulation of the conjugated inhibitors on transgenic mice with AD and the results indicated a major decrease of Aβ levels in the plasma and brain. Acetylcholinesterase inhibitors (AChEI) are commonly used in the treatment of AD [134]. The mechanism of action is through increasing the concentration of acetylcholine to improve cognitive symptoms. However, the major limitation of AChEI is frequent dosing that requires nursing of patients. Ideal therapies could provide long-term controlled drug delivery. Donepezil (Aricept^®^), a tablet preparation, is administered once daily. Rivastigmine (Exelon^®^), available as a capsule or as a liquid is administered once or twice daily depending on the therapeutic goal [135]. Galantamine is also currently used for the treatment of AD. Results from clinical trials have suggested that galantamine may improve the cognitive performance in individuals with AD [136]. Research has shown that deterioration of cells that produce acetylcholine in the brain affects thought processes. Galantamine is thought to work in two ways by inhibiting AChEI and may also stimulate nicotinic receptors in the brain to release additional acetylcholine [136]. Reminyl^®^ is available as an oral tablet of galantamine that requires twice daily administration [137]. A study conducted earlier by Schneider, [138] revealed that galantamine consistently failed to show statistically significant treatment effects at doses of 8mg/day and produced adverse gastrointestinal symptoms similar to other AChEI with increased dosages.

Memantine is also used for the management of AD, particularly for senile dementia [139]. The majority of current drugs that treat AD, such as galantamine, inhibits acetylcholinesterase that is responsible for the metabolism of the brain neurotransmitter, acetylcholine. However, memantine functions differently in that it inhibits N-Methyl-D-Aspartate (NMDA) receptors that underlie the degeneration of cholinergic cells (1) that are essential for healthy brain function. Over stimulation of NMDA receptors is referred to as excite-toxicity which is a common pathway for most neurological disorders [139,140].

Recent attempts were to target M_1_-muscarinic receptors intended for cognitive enhancement with the efficient lowering of Aβ. However, the non-selective nature of these compounds in targeting M_1_-receptors resulted in dose-limiting side-effects, precluded further development. Bale, [86] reported that muscarinic receptor modulation is a potential therapeutically viable approach and should be further explored. Rosenberg, [141] explored the area of gene vaccination to prejudice the immune response to Aβ. The gene-gun-administered genetic immunization with the Aβ(42) gene proved to be effective in eliciting humoral immune responses without a significant T-cell-mediated immune response to the Aβ peptide. This immunotherapeutic approach could be a breakthrough in the immunotherapy of AD [141]. The recent progress in defining the genotype-phenotype relationships in familial AD is a growing consensus that the most effective treatments for AD may be those that interrupt an obligatory early step that occurs before a progressive cascade of cell-damaging events. A summary of neuroactives that are currently being tested is listed in Table 1.

In AD senile plaques of Aβ are deposited and neuro-inflammation due to innate immune responses against Aβ is thought to cause degeneration of synapses and neural processes [142]. Earlier studies discovered that immunization of amyloid precursor protein (APP) in transgenic mice with Aβ reduced senile plaques and prevented new deposits of Aβ [143]. It was also found that antibodies to Aβ had a similar effect. Since immunized mice showed improvement of cognitive functions, an active immunization method was tested in humans. However, the trial was halted due to sub-acute meningo-encephalitis as a result of T-cell mediated autoimmune responses evolved as a side-effect [142]. A safe oral Aβ vaccine using adeno-associated virus vector has been developed which has shown to elevate antibodies and maintaining high titres over 6 months. APP transgenic mice and old monkeys have shown significant reduction of amyloid burden without any side-effects. Since Th1 immune responses were suppressed in the gut immune system, it is thought that immunization is a promising way to treat and prevent AD [142–144].

## 4. Overview of Parkinson’s Disease

James Parkinson firstly reported Parkinson’s disease (PD), in 1817 (Hoxton, London) as “the shaking palsy”. This is the second most common neurodegenerative disorder after AD. The etiology of PD is still being researched with few speculations as to the cause of the sporadic idiopathic Parkinson’s syndrome. The world prevalence of PD is approximately 150 individuals per 100 000 and increasing rapidly in those over 70 years of age [145]. Risk factors include demographics such as age, sex, race and geographical location. For instance, there is a greater frequency of PD in Europe than in North America. Older Caucasian males in industrialized regions are at a greater risk of developing PD than those in less industrialized regions. Exposure to diminutive doses of methylphenyl-tetrahydropyridine (MPTP), and previous exposure to viral encephalitis lethargica also present a great risk. There is startling evidence that PD is less prevalent in tobacco smokers than in lifelong abstainers [145]. Thus far, research has concentrated on genetics, environmental toxins, endogenous toxins and viral infection. The key features of PD are tremor, muscular rigidity, bradykinesia, postural instability and micrographia [145]. Atypical and typical parkinsonian syndromes are categorized according to the neuronal damage caused and the resultant clinical symptoms as described in Table 2.

### 4.1. The neuropathology of Parkinson’s disease

The neuropathological hallmark of PD is the progressive degeneration of dopaminergic neurons in the *substantia nigra pars compacta* of the brain in addition to astrocytic gliosis and the presence of numerous other neuronal systems, associated with widespread occurrence of intracytoplasmic α-synuclein positive inclusions known as Lewy bodies (LBs) and Lewy neurites of neuronal cells [146]. The ubiquitous protein α-synuclein is also involved in the pathogenesis of PD and comprises protein filaments of ubiquitin and α-synuclein that are the primary constituent of LBs. Aggregated α-synuclein binds the proteasome and potently inhibits proteasomal activity [147,148]. Schultz, *et al.* [149] demonstrated a correlation between α-synuclein and dopaminergic neurotransmission. The genetic involvement in PD is still a controversial topic. Mutation of the three genes, namely α-synuclein, a pre-synaptic terminal CNS lipophilic protein comprised of 140-amino acids, parkin and ubiquitin C-terminal hyrolase L1 have been identified as the potential cause of familial PD. However, the major constituent of LBs is said to be α-synuclein [150]. Mutation of α-synuclein on chromosome 2p13 and ubiquitin carboxyl-terminal hydroxylase L1 (UCHL1) on chromosomes 4p14-16.3, account for familial forms of PD particularly the autosomal dominant type [151,152]. Although only few families have thus far been identified with mutation of these genes, tau has been identified as a causative gene for frontotemporal dementia and Parkinsonism [153]. The onset of PD is typically during the fifth or sixth decade of life. However cases from as early as 20–40 years have been observed, where the disease is termed juvenile Parkinsonism.

In addition, studies showed the onset of lesions in the dorsal motor vagals nucleus and related medullary nuclei, constituting the “gain setting system” of the lower brainstem, and the olfactory system with ascending progression to the ceruleus complex, magnocellular nuclei of the basal forebrain system, substantia nigra compacta (SNc) and subnuclei of thalamus amygdala, and the allocortex, with inconsistent involvement of the isocortex [154,155]. The initial lesions in the lower brainstem and olfactory system may explain initial autonomic and olfactory impairment that may precede the somato-motor dysfunctions. The pattern of fully developed lesions may explain the complex somatomotor, autonomic, and behavioral symptoms. The major clinical subtypes of PD showed specific lesion patterns with neuropathophysiological and therapeutic relevance [155]. Akinesia-rigidity was significantly correlated with SNc neuron loss (ventrolateral part) and dopamine loss in the posterior striatum. The onset of clinical motor symptoms occurred with approximately 50% of SNc neuron loss and a reduction of striatal dopamine uptake and dopamine transporter loss by 56 – 80% [156,157]. Reduced dopaminergic input to the putamen caused an increased activity of the GABAergic indirect striatal efferent loop via SNr and medial globus pallidus (Gpi) leading to increased inhibition of the thalamocortical motor loop with reduced cortical activation [156]. Disinhibition of the indirect pathway leads to hypoactivity of external globus pallidus (Gpe) and subthalamic nucleus (STN) while increased excitatory drive from hyperactive STN may sustain progression of neurodegeneration in PD [158]. Increased GAGAergic activity reduced by L-dopa treatment disappeared in the course of disease and may induce changes in NMDA receptors and glutamatergic synapses, favoring motor complications [159]. The uncoupling of receptor systems is a major cause of drug resistance and adverse L-dopa effects [160]. The tremor-dominant type showed less severe neuronal loss in the lateral SNc, and additional damage to the retrorubral A-8 field projecting to the matrix of dorsolateral striatum and ventromedial thalamus [161], influencing the striatal efflux via SNr and the thalamus to prefrontal cortex [162]. PETand fMRI studies suggested increased activity of the ventral thalamic projections (VIM) and of cerebellar connections [163] having considerable implications for stereotactic treatment of PD.

**Figure 3.**
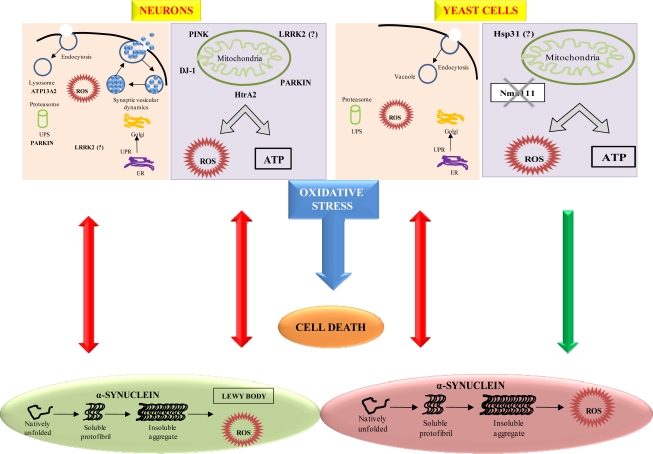
Molecular mechanisms leading to cell death in neurons and the yeast PD model (Adapted from: Winderickx *et al.* [164]).

Further recent elucidation of the molecular mechanisms implicated in PD has been demonstrated by Winderickx *et al.* [164]. The study has indicated that the yeast *Saccharomyces cerevisiae* is a suitable model to decipher molecular mechanisms involved in a variety of NDs caused by aberrant protein folding such as in PD. Among the 6 genes distinctly linked to familial PD, mutations in α-synuclein and LRRK2/dardarin resulted in autosomal dominant forms of PD, while mutations in parkin, DJ-1, PINK1 and ATP13A2 are implicated in autosomal recessive forms of PD. The association of other genes, such as the ubiquitin carboxy-terminal esterase L1, UCH-L1, the mitochondrial serine protease Omi/HtrA2 or the synaptic protein Synphilin-1, was less apparent. In Figure 3, Winderickx *et al.*, [164] depicted the major pathways involved in α-synuclein-mediated cell death, namely α-synuclein aggregation, obstruction of vesicular traffic and protein degradation, and mitochondrial dysfunction. In both neurons and yeast cells, aggregation of α-synuclein occurring simultaneously with reactive oxygen species (ROS) formation *in vitro*, was reported to hamper vesicle dependent processes, such as endocytosis and endoplasmic reticulum-to-Golgi traffic, to induce the unfolded protein response (UPR) and to block the ubiquitin–proteasome system (UPS). Sustained induction of the UPR and impairment of the UPS are purported to trigger the production of ROS. Furthermore, α-synuclein protofibrils cause vesicle permeabilization leading to disrupted homeostasis of dopamine (DA) metabolism, increased cytoplasmic DA levels, and enhanced oxidative stress in dopaminergic neurons. The production of ROS as described has adverse effects on mitochondrial function, and conversely, the oxidative stress induced by mitochondrial dysfunction is known to affect the UPS. In addition, a direct physical interaction of α-synuclein with the outer membrane of mitochondria was demonstrated and proposed as an alternative link explaining mitochondrial dysfunction in dopaminergic neurons. Finally, increased oxidative stress enhances the aggregation of α-synuclein. Figure 2 also depicts the various PD-associated proteins and their homologous counterparts in yeast, i.e. DJ-1 and its homologue Hsp31 as well as HtrA2 and its orthologue Nma111. Although Winderickx *et al.*, [164] reported the involvement of HtrA2 and DJ-1 in PD through yeast studies, the results were unable to confirm the role of Nma111 in α-synuclein-mediated apoptotic cell death and a protective function of Hsp31 have not yet been demonstrated. The role of LRRK2 in neuronal loss in PD is not yet known.

Furthermore, there is striking evidence on the anomalous accumulation of iron in the brain of PD patients, with the highest concentration in the substantia nigra, globus pallidus, red nucleus, caudate nucleus, putamen, substantia nigra pars compacta, microglia, as well as in association with neuromelanin. The level of iron within the brain increases with age, and the increased iron levels have been observed in several NDs. Neuroscientists have acknowledged the degenerative effects of free iron on neurons in the brain and its relation to the progression of PD [165,166]. Redox-active iron constitutes part of LBs. Iron and free radical generators such as DA and hydrogen peroxide are thought to augment the generation of toxic ROS and the aggregation of inert α-synuclein to toxic aggregates [167]. Insight on a few biochemical experiments have exposed the complexation between nicotine and free iron during the electrochemical actions of the Fe^2+^/Fe^3+^ redox couple [168].

It is well known that α-synuclein is broadly expressed in the brain, where it interacts with membranes, vesicular structures, and a variety of other proteins. In mammalian cells α-synuclein has been reported in the nucleus, cytosol, associated with membranes and, in diseased brains, in large cytoplasmic inclusions LBs [169]. Synucleinopathies are classified as protein-misfolding disorders [169]. Given the strong conservation of protein folding, membrane trafficking, and protein QC mechanisms between yeast and higher eukaryotes, Outeiro and Lindquist, [169], used *Saccharomyces cerevisiae* to uncover and establish basic aspects of both normal and abnormal α-synuclein biology. In order to explore α-synuclein dynamics in living cells, they created an α-synuclein-GFP (green fluorescent protein) fusion that was not subject to proteolysis in yeast cells as related fusions have been in mammalian cells [170]. By integrating the construct into the genome under the control of a galactose-inducible promoter allowed routine manipulations in the absence of α-synuclein expression. Upon induction with galactose, wild-type (WT) α-synuclein-GFP localized at the plasma membrane and a smaller quantity accumulated in the cytoplasm. Compared with other GFP fusion proteins, α-synuclein did not localize in mitochondrial or nuclear membranes. Thus, reminiscent of its selectivity for membranes with particular lipid compositions *in vitro*, α-synuclein had a high intrinsic selectivity for particular cellular membranes *in vivo*. Results from this study in conjunction with others have revealed that the approach is flexible and may provide an opportunity to analyze the molecular pathways underlying normal **α**-synuclein biology and the neuropathogenic consequences of its misfolding [170,171].

### 4.2. Clinical and therapeutic findings on Parkinson’s disease

The neurochemical effect is a decline in DA concentrations in the basal ganglia. The clinical signs of PD include mass loss and gastrointestinal symptoms such as indigestion and constipation due to the α-synuclein pathology in the autonomic nerves and ganglia. At present, the diagnosis of PD depends on recognizing clinical patterns as there are no viable laboratory-based diagnostic tests. Positron Emission Tomography (PET) is only used in research and conventional imaging is unhelpful [172]. Medical science lacks an accurate blood or imaging diagnostic test for PD, though tests can exclude other conditions. Diagnosis is based on an evaluation of symptoms best accomplished by PD medical specialists. The progression of PD varies and therefore treatment is individualized and focuses on relieving disabilities while minimizing side-effects caused by the drugs administered. While there is no cure, therapies can minimize symptoms and maximize the function and quality of life of those afflicted with PD. Even though PD is incurable and drugs are unable to alter the cause of the disease, levodopa (L-dopa) and other DA agonists are used in an attempt to restore DA activity. DA does not cross the BBB and has no therapeutic effect if administered systemically. However, L-dopa, the gold standard treatment for PD and an immediate precursor of DA, penetrates the BBB where it is decarboxylated to DA [173, 174]. L-dopa is usually combined with a decarboxylase inhibitor, carbidopa or benserazide, to reduce the peripheral conversion of L-dopa to DA thus reducing the peripheral side-effects. L-dopa remains the most effective treatment for the slowness of movement, increased muscle tone and tremor that are typical of PD [175].

Conversely, the inhibition of decarboxylase is associated with O-methylation of L-dopa leading to poor therapeutic responses. This further requires a catechol-o-methyltransferase inhibitor (COMT) such as entacapone to inhibit the reaction and reduce the end-of-dose fluctuations of L-dopa. Other directly acting DA receptor agonists include drugs such as bromocriptine, lisuride, pergolide, cabergoline, pramipexole and ropinirole acting principally on D_1_ receptors located in the zona compacta of the substantia nigra, D_2_ receptors found on striatal neurons, and D_3–5_ receptors [176]. These drugs are used as an alternative or an addition to L-dopa therapy. Apomorphine, a potent D_1_ and D_2_ agonist is administered subcutaneously by metered-dose infusion to level the fluctuations in response to L-dopa. Woitalla *et al.* [177] demonstrated the safety, tolerability and effectiveness of a transdermal formulation of lisuride that offers a promising therapeutic modality to manage the frequency and intensity of the motor fluctuations of PD. Other approaches of treatment include the use of selective monoamine oxidase-B (MAO-B) inhibitors such as rasagiline, a drug demonstrating neuroprotective properties that are independent of its MAO inhibitory activity [178].

Supplementary agents that are also used include antioxidants such as vitamins C and E that act as neuroprotectants [179]. Tissue transplantation of fetal or autologous DA containing adrenal medulla and glial cell-line neurotrophic releasing factor (GDNF) into the cerebral ventricles or basal ganglia failed to show any major clinical improvement. A group of scientists have recently applied the technique of introducing copies of genes into the brain to enhance the production of neurotransmitters such as DA. Candidate genes included tyrosine hydroxylase and GTP cyclohydroxylase I. Genes for neurotophic factors or anti-apoptotic agents can be used as a neuroprotective approach [180]. Thus, this research showed promise for the treatment and possible cure of PD. However the safety and efficacy of this approach requires further comprehensive tests to be conducted.

**Figure 4.**
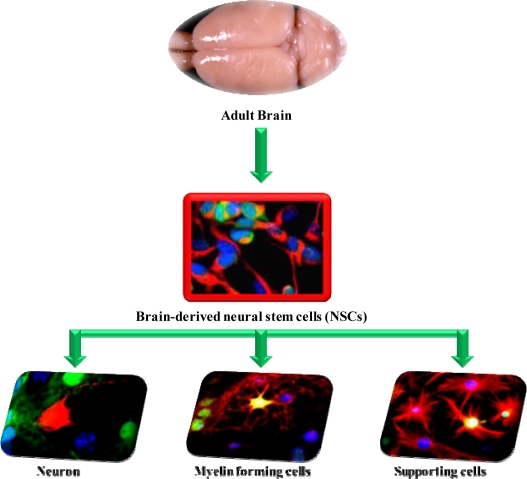
Depiction of adult neural stem demonstrating their intrinsic potential to generate cell types of the brain and spinal cord (Adapted from: Karimi and Eftekharpour, Fehlings lab, McEwan Centre for Regnerative Medicine; www.mcewencentre.com/res_prog_scnd.asp, [182]).

Clinical studies on cell transplantation as an alternative therapy for PD have been conducted since the early 1980s. Stem cells are used as a therapeutic alternative for PD. The current leading scientific explanation is that stem cells may surrogate the damaged cell population (Figure 4). In an attempt to increase the generation of DA in the brain, non-neuronal cells were transplanted, both without genetic manipulation and with genes like tyrosine hydroxylase, in animal models with PD [181]. The limitation of this study was due to the cells’ non-neuronal origin, leading to uncontrolled development of these cells. Transplanted neuronal cells are likely to survive and grow into neurons with functional synapses and lead to meaningful clinical improvement. Fetal dopaminergic cells originating in the ventral mesencephalic tissue were acquired for such experiments. Transplanted cells are anticipated to be only functional if they have the same capacity as the original neurons, such as synthesizing, releasing and metabolizing neurochemicals. The discovery of this self-renewing phenomenon of embryonic and adult stem cells to produce differentiated tissues confers a promising future for tissue-replacement in NDs.

O’Brien *et al.* [183] conducted a study to examine the extent and pattern of DA transporter loss using iodine I 123-radiolabeled 2β-carbomethoxy-3β-(4-iodophenyl)-*N*-(3-fluoropropyl) nortropane (FP-CIT) with Single-Photon Emission Computed Tomography (SPECT) in DLBs compared with other dementias and to assess its potential to enhance a differential diagnosis. They established that significant reductions (p < 0.001) in FP-CIT binding occurred in the caudate and anterior and posterior putamens in subjects with DLB compared with subjects with AD and controls. Transporter loss in DLBs was of similar magnitude to that seen in PD, but with a flatter rostrocaudal (caudate-putamen) gradient (p = 0.001), while the greatest loss in all three areas was observed in those who had PD and dementia. Both region of interest analysis and visual ratings provided superior separation between DLBs and AD (region of interest: sensitivity, 78%; specificity, 94%; positive predictive value, 90%) but not among subjects with DLB, PD, and PD with dementia. It has thus been concluded that DA transporter loss can be detected *in vivo* using FP-CIT SPECT in DLB [183,184].

### 4.3. The symptomatic treatment of Parkinson’s disease

In PD, cells are destroyed in the substantia nigra of the brain stem that transmits fibers to the corpus stratia which has DA-releasing cells. A challenge is that when symptoms develop, patients have already lost 80–90% of DA-producing cells [185]. Since certain drugs and other conditions can cause Parkinson’s-like neurologic symptoms, the misdiagnosis of PD is frequent [185]. Besides L-dopa, the non-ergoline D_2_-agonists, ropinirole, is also used for the symptomatic treatment of PD [186]. The efficacy of ropinirole was investigated in placebo-controlled studies by Brooks *et al.*, [187]. One study used ropinirole as monotherapy in early PD while others employed it as an adjunct to L-dopa in patients who experienced fluctuations in motor response. Ropinirole therapy over 12 weeks was found to be effective for symptomatic therapy in both patient groups. Unlike other DA agonists, CNS side-effects were of the same magnitude as for patients receiving a placebo [187]. The antiviral agent, amantadine, is also used for the symptomatic treatment of PD for over 30 years [188,189]. Studies have suggested that amantadine may have a neuroprotective effect in PD mediated through N-methyl-D-aspartate (NMDA) receptor antagonism, DA-uptake blockade or other mechanisms [188,189]. Selegiline, a monoamine oxidase-B (MAO-B) inhibitor has also been used for the symptomatic treatment of PD. Selegiline selectively and irreversibly inhibits intracellular and extracellular MAO-B and therefore reduces or delays the degradation of DA while also inhibiting the reuptake of DA from the synaptic cleft [190]. Furthermore, it has been observed that the addition of selegeline to L-dopa may allow a reduction of the L-dopa dose [191,192]. However, selegiline may also potentiate the side-effects of L-dopa such as dyskinesias and psychiatric tendencies [193].

The anticholinergic drugs such as biperiden, procyclidine, orphenadrine, benzhexol, and benztropine are used to improve the tremor and stiffness to a greater degree than akinesia and are overall mildly effective [194]. Due to a peripheral parasympathomimetic action, side-effects such as glaucoma, dryness of the mouth, blurred vision, urinary retention, and constipation may occur. Since anticholinergics have a relatively high potential for causing or worsening confusional states and impairing concentration, they are used cautiously in the elderly [195]. The addition of a COMT inhibitor as adjunctive therapy to L-dopa with either carbidopa or benserazide reduces the peripheral metabolism of L-dopa. This prolongs the plasma half-life of L-dopa and increases the quantity available in the brain [196,197]. COMT inhibitors are however associated with side-effects such as potentiation of dyskinesias, nausea, diarrhea, discoloration of the urine and sleep disturbances.

In addition, results of the first placebo-controlled, multicenter clinical trial of the compound Coenzyme Q_10_ (CoQ_10_) undertaken by Shults and coworkers [198], suggested that CoQ_10_ may slow disease progression in patients with early-stage PD. The phase II study enrolled 80 subjects with early PD who did not require treatment for their disability to determine if CoQ_10_ is safe and if it has the capacity to reduce the rate of functional decline in PD. The subjects underwent evaluation with the Unified Parkinson Disease Rating Scale (UPDRS) at the screening, baseline, and were followed up for 16 months or until disability requiring treatment with L-dopa had developed. The primary response variable was the change in the total score on the UPDRS from baseline to the last visit. Results have shown that CoQ_10_ was safe and well tolerated at dosages of up to 1200mg/d. Less disability developed in subjects assigned to CoQ_10_ than in those assigned to the placebo, and the benefit was greatest in subjects receiving the highest dosage. CoQ_10_ appeared to slow the progressive deterioration of function in PD. However the results need to be confirmed in a larger group of subjects [198].

Embryonic/fetal cell transplantation has been introduced, as a treatment option for PD, based on the hypothesis that embryonic or fetal neuronal cells would regenerate the damaged neuronal tissue in patients with PD, thereby reducing their symptoms and/or slowing disease progression. Dopaminergic neuronal cells are obtained from the brainstem of aborted embryos/fetuses aged 6–15 weeks post-conception. The cells are transplanted into the brain of a patient with PD. Other sources of dopamine neuronal cells currently under clinical investigation include (embryonic, fetal, and adult) stem cells, adrenal medullary tissue, retinal pigment epithelium, carotid body cells, and autologous and xenogeneic neuronal tissue. The majority of studies evaluated involve human fetal mesencephalic tissue. The primary outcome measure in all studies was changes in PD motor function. Symptom severity was generally assessed with the Unified Parkinson’s Disease Rating Scale (UPDRS), assessed in the “on-drug” and/or “off-drug” state following an overnight withdrawal of anti-PD medication [199,200,201]. In a prospective 24-month double-blind controlled trial by Olanow and coworkers [202], patients with advanced Parkinson’s disease were randomized into one of three groups [202]. One group received bilateral transplantation with one donor per side, one group received bilateral transplantation with four donors per side and the last group received a placebo procedure. Changes in UPDRS scores from baseline to 24 months post-surgery, assessed in the “on-drug” and “off-drug” state, served as the primary outcome measures; secondary outcome measures included dopamine production in the patients’ brains and motor function as recorded in a patient self-maintained diary. There was no significant overall treatment effect. Patients in the placebo and one-donor groups showed deterioration whereas those in the four donor group showed some improvement. Fifty-six percent of the transplanted patients developed dyskinesia that persisted after overnight withdrawal of dopaminergic medication. Therefore the authors concluded that fetal nigral transplantation cannot currently be recommended as a therapy for Parkinson’s based upon these results [202].

## 5. Overview of Amyotrophic Lateral Sclerosis

Amyotrophic lateral sclerosis (ALS) is a progressive degenerative motor neuron disease characterized by weakness in limb and bulbar muscles with atrophy, spasticity, weight loss and ultimately respiratory failure. The prevalence of ALS is approximately two per 100,000 per annum, and it is estimated that there are about 25,000 prevalent patients in North America [203]. The onset of ALS may be subtle that the symptoms are frequently overlooked and usually occur between the fourth and sixth decade of life. However, any age group from all races and ethnic backgrounds can be affected. Men are at a higher risk than women. Also known as Lou Gehrig’s disease, ALS is an age-dependent rapidly progressive disorder of motor neurons with both sporadic and familial forms that is ultimately fatal. The disease is virtually always fatal and approximately 50% of patients die within 3–4 years after the onset of symptomatic weakness. There is a combination of upper motor neuron and lower motor neuron abnormalities, and relentless and nearly linear progression of impaired function in almost all patients. Approximately 95% of ALS is classic sporadic [204–210]. Sporadic ALS has been associated with ‘susceptibility’ genes as the neurodegenerative effect of these genetic changes probably depends on interactions with other prevalent, low penetrant genetic defects and/or with environmental risk factors. Familial forms with Mendelian inherited ALS are observed in approximately 5 – 10% of patients and are clinically and neuropathologically indistinguishable from sporadic ALS [204–207]. Motor neurons located in the brain, brainstem, and spinal cord serve as controlling units and communication links between the nervous system and the voluntary muscles of the body. In ALS, both the upper and lower motor neurons degenerate and neural communications with the muscles are impeded. ALS results in progressive muscle weakness, atrophy, fasciculations and cramps. The respiratory muscles in the diaphragm and chest wall are also affected, and patients lose the ability to breathe without ventilatory support. Patients also face an increased risk of pneumonia during later stages of ALS. Death after 3–5 years is mostly from respiratory failure. However, approximately 10% of ALS patients survive for 10 or more years [211].

### 5.1. The neuropathology of Amyotrophic Lateral Sclerosis: The role of inflammation

**Figure 5.**
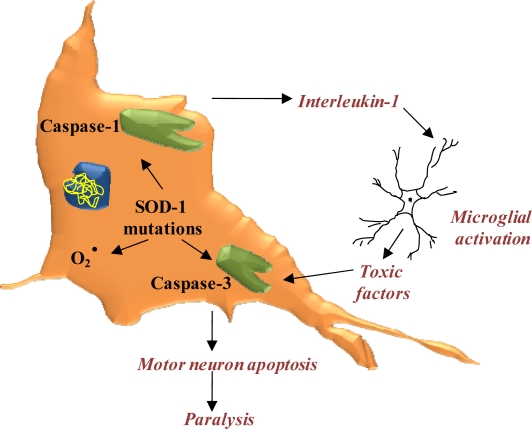
Schematic of SOD-1 mutations activating cell death pathways in familial Amyotrophic Lateral Sclerosis (Source: Yuan and Yankner, [16]).

The neuro-inflammatory genes implicated in ALS include TNF-alpha, IL- RA, CD86, CD200R and Groalpha [212]. The inflammation neuropathology is complemented by glutamate neurotoxicity, oxidative stress, aberrant metal ion regulation, apoptosis, as well as abnormal microglial function on G93A SOD1 that results in neurodegeneration [213]. Figure 5 depicts SOD-1 mutations that may activate caspase-1 and caspase-3, thus potentially increasing free-radical generation, leading to motor neuron apoptosis. The activation of caspase-1 leads to interleukin-1 production, which can induce a local microglial inflammatory response and increase the number of neurons affected [16].

### 5.2. Clinical and therapeutic findings on Amyotrophic Lateral Sclerosis

No single test can provide a definitive diagnosis of ALS, although the presence of upper and lower motor neuron signs in a single limb is strongly suggestive. Diagnosis of ALS includes signs and symptoms of both upper and lower motor neuron damage that cannot be attributed to other causes. The clinical representation is a progressive spastic tetraparesis or paraparesis including fasciculations, cramping, stiffness of muscles, muscle weakness, slurred and nasal speech, and difficulty chewing or swallowing progressing to atrophy. The early symptoms depend on the region that is damaged first. Atrophy eventually spreads to other regions of the body as the disease progresses and leads to mass loss, dysphagia, dysarthria, muscle spasticity, and hyper-reflexia with an overactive gag reflex. The Babinski’s sign is also an indicator of upper motor neuron damage. Diagnostic tests include electromyography (EMG), for electrical activity in muscles. EMG findings may complement the diagnosis of ALS. Another common test measures nerve conduction velocity (NCV) for peripheral neuropathy or myopathy as a differential diagnosis for ALS. A magnetic resonance imaging (MRI) scan may be used for assessing the brain and spinal cord. A few researchers have investigated the role of glutamate, a brain neurotransmitter, in motor neuron degeneration. It has been established that ALS patients have elevated levels of glutamate in serum and spinal fluid [214–217]. *In vitro* studies have demonstrated that neurons begin to degrade when exposed for long periods to excessive quantities of glutamate.

However the mechanism of glutamate build-up is still to be determined. Furthermore, autoimmune responses have also been implicated in motor neuron degeneration in ALS [218]. The cures for ALS is not known and successful pharmacotherapy intended to slow, seize or repeal the disease progression, remains a challenge [212]. However, the US FDA has approved riluzole (Rilutek^®^) as a drug for treating ALS [219]. Riluzole is able to reduce the damage to motor neurons by decreasing the release of glutamate. Clinical trials with ALS patients showed that riluzole does not reverse the damage, but prolongs survival by several months, mainly in those with difficulty swallowing and prolongs the time before ventilatory support is needed. Supportive care from a multidisciplinary team of health care professionals such as general practitioners, pharmacists, physiotherapists, occupational therapists, speech therapists, dieticians, social workers, as well as home care and hospice nurses is vital. Drugs to help reduce fatigue, ease muscle cramps, control spasticity, and reduce excess saliva and phlegm may be administered. Pharmacists may provide advice on the proper use of medications and monitor a patient’s prescription to avoid risks of drug interactions. Potential therapies for ALS being investigated include antioxidants and neurotrophic factors to prevent neurodegeneration. Recent advances in research have also identified an ubiquinated protein that is commonly found in the majority of ALS cases. In healthy nerve cells the protein, TDP-43, is typically localized in the nucleus. However, in affected nerve cells, TDP-43 is only found in the cytoplasm. The cytoplasm is located outside of the nucleus and is the primary site for chemical activity in the cell. The accumulation of abnormal TDP-43 in this area of the cell is speculated to cause a loss in cellular function (due to the absence of normal TDP-43 in the nucleus), thereby impairing the viability of the affected nerve cells [220,221].

### 5.3. The symptomatic treatment of Amyotrophic Lateral Sclerosis

Since patients with ALS have symptoms of progressive muscle weakness, disturbed speech and swallowing, and in the terminal phase respiratory weakness, treatment options may be introduced when the patient is emotionally capable and prior to dysarthria [222]. Cramps, pathological crying or laughter, spasms, and spasticity are treated symptomatically. Sialorrhoea which results from ALS is caused by difficulty in swallowing and is normally accompanied by a danger in aspiration. It can be halted by the use of medication, radiotherapy or by injecting botulin toxin into the salivary glands. Dysphagia results in mass loss which can be prevented by eating frequent small meals or if necessary performing a percutaneous endoscopic or radiological gastroscopy. The terminal phase of ALS is associated with restlessness, anxiety, pain and dyspnoea and it requires a careful and well co-operated multidisciplinary palliative care [222]. Minimal research progress has been made in terms of developing new strategies for the symptomatic management of ALS.

The neuroactive drug, riluzole that reduces glutamate release from nerve terminals has been approved for treatment of patients with ALS in most countries. However, questions persist regarding the clinical utility of riluzole due to its high cost and modest efficacy. Miller and coworkers [223], reviewed the efficacy of riluzole in prolonging survival, and in delaying the use of surrogates (tracheostomy and mechanical ventilation) to sustain survival in patients with ALS. In their study 100mg of riluzole was administered daily and was found to be reasonably safe and potentially prolonged the median survival of ALS patients by approximately 2 – 3 months. In addition evidence from 4 randomized clinical trials involving 1477 patients with ALS was also examined and concluded that patients taking riluzole probably survive longer than patients taking the placebo. However, the beneficial effects were modest and the drug was found to be rather expensive [223]. Further studies are required, especially to determine whether patients treated earlier or older, more advanced patients with longstanding disease derive the same benefit. In terms of side-effects the most frequent are nausea and asthenia and liver function requires monitoring [223].

## 6. Overview of Huntington’s Disease

Huntington disease (HD) is a progressive heredo-neurodegenerative disease manifested by chorea and other hyperkinetic (dystonia, myoclonus, tics) and hypokinetic (parkinsonism) movement disorders. In essence, HD is an autosomal dominant inheritance with complete penetrance, caused by mutation associated with trinucleotide repeat expansion repetition at the IT15 gene on the short arm of chromosome 4p which encodes a 349 kD huntingtin protein [224]. It is also known as a polyglutamine disease [225]. The macroscopic signs of HD are the bilateral marked atrophy of the putamen and the head of the caudate nucleus. At late stages of HD there is a tendency of small amino-terminal fragments with mutants to self-aggregate with an altered conformation. The onset of HD is usually in the third or fourth decade of life, although it can also attack children and the elderly. Worldwide prevalence of HD is approximately 5 in 100,000 persons [224].

### 6.1. The neuropathology of Huntington’s disease

**Figure 6.**
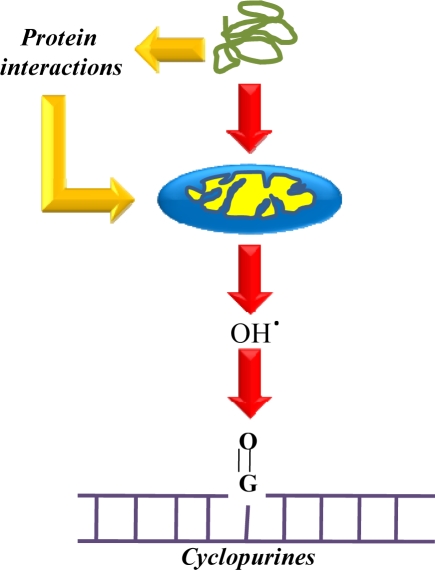
Schematic depicting a pathway of oxidative damage in HD (Source: Trushina and McMurray, [232]).

A growing number of NDs including HD have been found to belong to the group of CAG triplet repeat disorders [226]. These disorders are caused by an elongated CAG repeat located in the coding region of the respective genes, which is translated into a polyglutamine tract. The mechanism by which CAG repeats elongate is currently unknown and therefore is the subject of intensive investigation. Characteristic features of CAG repeat disorders are autosomal dominant inheritance, late onset, selective neurodegeneration, genetic anticipation, a pathological threshold at which the mutation becomes virulent, and an inverse correlation between CAG repeat length and age at disease onset [227–230]. Genetic mutation discovered in the chromosome 4 (4p16.3), in conjunction with a variable expansion of a trinucleotide CAG-repeat sequence, encoding polyglutamine, in axon 1 in huntingtin gene leads to the translation of an extended glutamine sequence in huntingtin, a predominantly cytoplasmic protein that is found in neurons throughout the brain [231]. The N-terminal fragments of mutant huntingtin subsequently aggregate in neurons causing progressive neuronal dysfunction and fatality. Adult HD sufferers have CAG expansions of approximately 40 – 55 repeats, whilst childhood cases are associated with up to 70 repeats. The oxidative DNA damage observed in HD can be caused directly and/or indirectly through altered mitochondrial function [232]. In the hereditary HD, expression of mhtt in HD can directly increase production of ROS and subsequent oxidative DNA damage through defects in mitochondria (Figure 6).

The main features of HD are sub-cortical dementia, choreiform movements including tics and grimaces, as well as altered moods. The neuropathogenesis begins with the degeneration of the basal ganglia and cerebral cortex with fractional loss of striatal neurons. There is marked loss of neurons in the caudate nucleus and putamen. Neurochemical transformations include a decline in concentrations of cholineacetyl transferase, an enzyme that synthesizes acetylcholine the striatum. There is also an increase in transglutaminase, an enzyme that catalyzes aggregates of huntingtin in the cortex, cerebellum and corpus striatum.

The specific progressive atrophy in these brain regions is associated with reactive astrocytosis [233]. Within the striatum there is selective loss of medium spiny γ-aminobutyric acid (GABA) neurons, which project into the pallidum forming the indirect striatopallidal pathway. Prior to cell death, neuronal dysfunction is manifested by abnormalities of dendritic endings. In addition to atrophy in the striatum, extensive neuronal cell loss also occurs in the deep layers of the cerebral cortex, white matter, hippocampus, amygdala, and thalamus [234]. Studies have shown that the mutation underlying HD is an unstable CAG trinucleotide repeat expansion in the first axon of the gene. It ranges from 6–37 units in healthy individuals, and 38–180 units in HD patients [227,235]. The CAG repeat is translated into a polyglutamine stretch, which is conserved in vertebrates, containing 7 glutamines in the mouse [229] and only 4 in the puffer fish, [230] but is absent from the *Drosophila* protein [236]. The predicted huntingtin protein sequence is highly conserved between human, mouse, and puffer fish, but showed no significant homology with other proteins in databases. The only functional motives that have been discovered are a putative leucine zipper and a HEAT repeat [237]. HEAT repeats consist of two hydrophobic α-helices and were found in proteins involved in cellular transport processes. Few studies have found huntingtin interacting protein–1 (HIP1) associated with the HEAT repeat [238]. However, whether this sequence motive is essential for protein-protein interaction is yet to be determined. HIP1 has been identified using the yeast two-hybrid system. The predicted amino acid sequence of HIP1 exhibits significant similarity to cytoskeleton proteins, suggesting that HIP1 and the huntingtin protein play a functional role in the cell filament networks and/or vesicle trafficking [239], which associates with the membrane cytoskeleton and plays a functional role in endocytosis [240]. Recently, colocalization of HIP1 and huntingtin with clathrin-coated vesicles in mammalian cells has been described, suggesting that both proteins also play a functional role in endocytosis in higher eukaryotes [241, 242]. This hypothesis is substantiated by the finding that huntingtin and its associated protein, huntingtin-associated protein-1 (HAP1), [243] may be transported along microtubules in axons [244]. In addition, direct binding of HAP1 with p150Glued of the dynactin complex, which is critical for retrograde movement of vesicles along microtubules, has been described [245]. Overall, these findings indicate that a protein complex consisting of the proteins HIP1, HAP1, and huntingtin is functionally involved in endocytosis and retrograde transport of clathrin-coated vesicles along microtubules. However, additional cell biology and biochemical studies will be necessary to address this hypothesis in more detail. The generation of HIP1 and SH3GL3 knockout as well as transgenic animal models may help elucidate the normal function of huntingtin and may also help to understand the key steps in the neuropathogenesis of HD.Giorgini and coworkers [246], focused on elucidating the molecular mechanisms for the development of newer therapeutic strategies for HD using a yeast model of mutant huntingtin toxicity. Conserved cellular pathways involved in mutant huntingtin toxicity and thus candidate drug targets for the treatment of HD have been identified. HD is caused by expansion of a polyglutamine tract in the protein huntingtin, which leads to its aggregation in nuclear and cytoplasmic inclusion bodies. Giorgini and coworkers [246] have identified 52 loss-of-function mutations in yeast genes that may enhance the toxicity of a mutant huntingtin fragment and have also identified 28 gene deletions that suppress toxicity of a mutant huntingtin fragment. The suppressors may play a role in vesicle transport, vacuolar degradation, transcription and prion-like aggregation. Among the most potent suppressors identified was Bna4 (kynurenine 3-monooxygenase), an enzyme in the kynurenine pathway of tryptophan degradation that has been linked directly to the neuropathophysiology of HD in humans by a mechanism that may involve reactive oxygen species. Their work provides new insights and is suggestive of a conserved mechanism of polyglutamine toxicity from yeast to humans and identifies new candidate therapeutic targets for the treatment of HD [246].

Vonsattel and coworkers, [247] explored the neuropathological classification of HD. In postmortem brain specimens from 163 clinically diagnosed cases of HD the striatum exhibited marked variation in the severity of neuropathological involvement. A system for grading the severity was established by Vonsattel and coworkers, [247] by macroscopic and microscopic criteria, resulting in five grades (0–4) designated in ascending order of severity. The grade correlated closely with the extent of clinical disability as assessed by a rating scale. In five cases of clinically diagnosed HD there were no discernible neuropathological abnormalities (grade 0), suggesting that the anatomical changes lag behind the development of clinical abnormalities. In eight cases, neuropathological changes could only be recognized microscopically (grade 1). The earliest changes were seen in the medial paraventricular portions of the caudate nucleus (CN), in the tail of the CN, and in the dorsal part of the putamen. Counts of neurons in the CN reveal that 50% were lost in grade 1 and that 95% were lost in grade 4. In addition, astrocytes were greatly increased in grades 2 – 4. These studies indicated that analyses of the CN in grade 4 may reflect mainly its astrocytic composition with a component of remote neurons projecting to the striatum. Due to the relative preservation of the lateral half of the head of the CN in grades 1–2, these regions would reflect early cellular and biochemical changes in HD [247].

### 6.2. The symptomatic treatment of Huntington’s disease

In HD, a variety of psychiatric and behavioral symptoms, along with cognitive decline, contribute significantly to the patient’s disability. Since there are no effective neuroprotective therapies that delay the progression of HD, symptomatic treatment remains the cornerstone of clinical management [248]. Several classes of drugs have been used to ameliorate the various symptoms of HD, including typical and atypical neuroleptics, DA antagonists, antidepressants, antiglutamatergic drugs, GABA agonists, antiepileptics, acetylcholinesterase inhibitors, and botulinum toxin [248]. Recently, surgical approaches including pallidotomy, deep brain stimulation and fetal cell transplants are being used for the symptomatic treatment of HD. Importantly is that the selected therapy must be customized to the needs of each patient in order to minimize the potential side-effects.

Although significant advances in identifying and clarifying the molecular and mechanistic pathways that lead to the progression of HD have been realized, effective pharmacotherapy remains a mystery and no treatment arrests the disease. Olanzapine 2.5 mg daily, a dopaminergic-serotonergic antagonist, has been suggested as an alternative therapy for HD [249,250]. Olanzapine 5 mg per day is administered with lofepramide 140mg daily for psychiatric improvement. The psychiatric manifestations were shown to improve in patients treated with a daily combination of 5 mg olanzapine and 1,500mg valproic acid [251].

## 7. Other Related Atypical Neurodegenerative Disorders

NDs not extensively reviewed in this paper include disorders such as Wilson’s disease (WD). Also known as hepatolenticular degeneration, WD is an autosomal recessive inherited disease that causes excessive copper accumulation in the liver or brain. Spontaneous mutation of the ATP7B gene located on chromosome 13, results in anomalous copper metabolism causing psychiatric and extrapyramidal symptoms [252]. Pick’s Disease (PkD) is characterized by the tau-positive Pick’s bodies. The symptoms of PkD are usually mistaken for AD and usually discovered during postmortems [253,254]. Multiple sclerosis (MS) that may be caused by genetic polymorphism (although controversial), results in CNS symptoms such as slurred speech, blurred vision and urinary incontinence [255,256]. Axonal loss is a major pathologic process responsible for irreversible neurologic disability in patients with multiple sclerosis (MS). Pathologic studies implicate inflammatory demyelination as a principal cause of axonal transection and subsequent axonal degeneration. Axonal degeneration caused by chronic demyelination in the absence of active inflammation may also contribute to progressive disability in the later stages of the disease. Studies using magnetic resonance spectroscopy suggest that axonal loss begins at the onset of the disease, and studies using MRI have documented brain atrophy in the earliest stages of MS [257]. Although these conditions are less common than AD, PD, ALS and HD, their severity and impact on society cannot be underestimated.

## 8. The Therapeutic Implications of Neurodegenerative Disorders

From a neurotherapeutic perspective, it is important to determine whether the early increases in neural activity are compensatory or neuropathogenic mechanisms, and to differentiate progression of the original neuropathogenic process from the emergence of co-pathogens and the age-related failure of compensatory mechanisms [258]. A more comprehensive understanding of the complexities encountered with NDs may facilitate the development of superior diagnostic and treatment modalities. Although a selection of the current treatments appear to improve the function of remaining neural circuits, their effect is limited and none seems capable of a permanent functional rescue [259,260]. This suggests that the target strategies employed are too few for the most critical derangements or the derangements are too advanced or dynamic for standard regimens [261]. It is also of great interest that several disorders, including AD and HD, are associated with an increased incidence of seizures as observed in previous studies in transgenic mice, whose neurons express human amyloid precursor proteins or mutant huntingtin, were prone to epileptic activity [262–265]. These and other observations suggest that the relationship between NDs and epilepsy should be further explored, particularly during the early stages and in susceptible sub-populations [38,41].

Drugs aimed at neurotransmitter receptors often fail due to signaling pathways that are disrupted by abnormal protein assemblies, creating a downstream block that cannot be overcome by upstream modulation. Encouragingly, experimental models are helping to unravel the molecular cascades through which abnormal protein assemblies may cause disruption [38,266,267]. A pertinent example is the recent demonstration that inclusion-body formation in HD does not harm but rather protects neurons against the mutant huntingtin protein. A few compensatory mechanisms may result in cell survival at the cost of network failure [268]. Thus far, studies have demonstrated that neural plasticity may result in a significant, though partial, recovery of neurological function after destruction of an important neuronal population [267].

Needless to say, is that NDs are chronic, but not static. For example, choline-acetyltransferase activity in the hippocampus is increased in mild cognitive impairment, which may precede or represent early AD, but is decreased in later stages of the disease raising questions regarding the timing for cholinergic-replacement therapy in AD [269,270]. Radiological imaging studies in patients with mild cognitive impairment have identified hyperactivation of hippocampal and neocortical regions that become hypoactive and atrophic with the progression to AD [271]. Similarly, neuronal activity in the globus pallidus appears to be increased during early stages and decreasing in advanced stages of PD. To facilitate the development of superior diagnosis and treatment modalities for NDs, these intricacies will have to be confronted with diligence.

## 9. Specialized Drug Delivery Strategies for the Treatment of Neurodegenerative Disorders

Pharmaceutical drug delivery technologies have shown that controlled release of old and new neuroactives may decrease the frequency of side-effects [272]. Biodegradable implant technology provides a platform for the delivery of neuroactives over several weeks or months in order to maneuver the BBB. Such technology is based on the use of bioerodible polymers and excipients, which have a proven record of safety and effectiveness in approved drug delivery and medical device products. The role of drug delivery technology is significant in the milieu of various therapies for NDs including neuroactives in development and the role of unique drug delivery systems. These novel methods of drug delivery will have an impact on pharmaceutical and neuro-clinical advancement. In their study, Vinogradov and coworkers [273], recommended a novel nanoscale network of crosslinked poly(ethylene glycol) and a polyethylenimine nanogel for the delivery of neuroactives such as oligonucleotides to the brain. This was an attempt to overcome the challenges posed by the BBB restricting the entry of neurotherapeutic molecules and to control drug delivery. Nanogels were able to bind and encapsulate spontaneously to negatively charged barriers and formed an aqueous dispersion of a polyelectrolyte complex with particle sizes less than 100nm that were transported across the BBB. In general, the work hinted at an optimistic future on nano-enabled drug delivery systems to the brain for NDs. Rice and coworkers [274], worked on strategies to overcome the p-glycoprotein-mediated efflux of paclitaxel (Taxol^®^) analogs, which are potential treatments for brain tumors and NDs.

Biodegradable thermoplastic polymers such as poly(lactic-co-glycolic acid) (PLGA) have been used to produce microparticles, for the controlled release of the AD drug, tacrine. Tacrine has a half-life of 2 – 3 hours, with gastrointestinal, cholinergic, and hepatic side-effects at high doses. The controlled drug delivery system was designed to help minimize side-effects by controlling the plasma levels of tacrine with sustained delivery [272]. This serves as evidence that polymer engineering by manipulating the physical and mechanical strength of polymers may enhance the therapeutic effectiveness of neuroactives.

**Figure 7.**
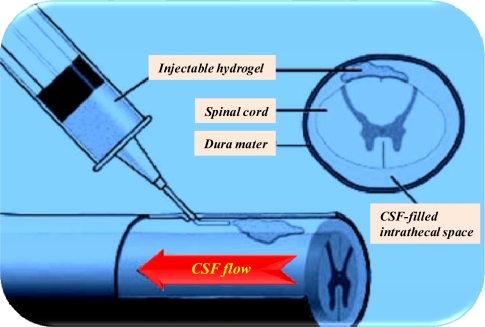
A minimally-invasive intrathecal drug delivery system for spinal cord injury repair (Source: Shoichet lab, McEwen Centre for Regenerative Medicine, [276]).

Tissue engineered devices may also be formed from biodegradable polymers and de-cellularized allografts. Cell therapy can be performed where growth factors and neuroactives may be incorporated into substrates to encourage cell attachment and function. Stem cells are also a potential therapeutic intervention. However, there are numerous controversies with the use of fetal sources of stem cells in a few developing countries. Stem cells can be genetically modified prior to transplantation for therapeutic interventions in NDs [43,275]. Tissue engineering in the spinal cord has implicated the implantation of scaffold material to guide cell placement and foster cell development. The scaffolds may also be used to deliver stem cells at the site of interest for enhancement of their regenerative potential. At the McEwen Centre for Regenerative Medicine, [276], efforts have been made to develop and test various three-dimensional scaffolds in experimental models of spinal cord injury (Figure 7). The scaffolds are intended to provide support to replace lost tissue or to act as guidance channels for neurons to regenerate beyond the site of injury. Ultimately they could serve as a platform for growth factors or neuroprotective drugs.

V1512 is an effervescent drug delivery system being developed for the symptomatic treatment of PD. It is a more soluble form of L-dopa comprising methyl ester L-dopa and carbidopa [277]. It is envisaged that V1512 will be absorbed more rapidly and consistently than conventional L-dopa preparations and thus would offer patients the potential of greater drug efficacy. The clinical efficacy and safety of V1512 has been established in a series of studies, which have shown evidence of a significantly more rapid onset of action and of reduced ‘off’ episodes (improved mobility). Clinical trials have also demonstrated a more reliable drug response in comparison with conventional L-dopa preparations. V1512 is in phase III clinical trials in which the reduction in total daily ‘off’ episodes will be used as a primary endpoint in the pivotal trials. Pharmacokinetic studies comparing plasma levels of V1512 with Sinemet^®^, a conventional and widely used L-dopa preparation, are also continuing [277].

Dopaminergic therapies which include L-dopa and DA-agonists remain the most distinct treatment of PD. With the exception of apomorphine injection which is a short-acting DA-agonist, there is no other widely available non-oral dopaminergic therapy [278]. Rotigotine is a lipid-soluble, non-ergot, D_3_, D_2_ and D_1_ DA-agonist that has demonstrated efficacy as an alternative therapeutic option in both early and advanced PD patients [278–280]. More importantly, it is uniquely formulated as a transdermal patch system allowing for continuous, once-daily administration and superior patient compliance. Preclinical and clinical trials have proven rotigotine to be a well-tolerated and an effective treatment for early-stage PD [278,280]. Rotigotine has also shown promise as adjunctive therapy with L-dopa for the treatment of advanced PD [279]. The side-effects of rotigotine have been found to be similar to those of other transdermal systems and DA-agoni**s**ts [280].

## 10. Conclusions

AD, PD, ALS and HD are complex neurodegenerative disorders. Their etiology is mostly unknown. However severe genetic mutations that lead to miscoding and aggregation in extracellular protein depositions have been observed. To date, no completely effective therapies have been developed. Research findings that implicate the commonalities in protein aggregations could insinuate that NDs share common or overlapping neuropathogenic mechanism(s) which could be targeted by synchronized therapeutic strategies. This review provides a model of how the integration of basic neuroscience, pharmacology and novel drug delivery technologies may warrant conquering diverse approaches for the treatment of NDs. Conceptualizing the molecular basis and neuropathology of NDs will offer a worthwhile enlightening understanding of NDs for a broader range of research scientists. Furthermore, future studies that will be able to confirm the neuropathogenic significance of reversible network dysfunction in NDs may make it possible to shorten clinical trial periods and to evaluate a greater diversity of neuroactive compounds. This could accelerate the pace of drug validation and offer obvious cost savings. This strategy may also facilitate the identification of effective combinations of neuroactives with distinct modes of action. Although such regimens are difficult to assess in clinical trials, they have proved useful in other multi-factorial diseases, such as epilepsy, hypertension and cancer, and will probably also be required for the effective treatment of NDs. Contemporary findings in novel therapeutic targets and treatments, to expose novel trails into drug delivery are also a significant part of this challenge.

## Figures and Tables

**Table 1.** Neuroactive classes that are currently being investigated for the treatment of Alzheimer’s disease.

**Class of Drug**	**Therapeutic/Pharmacological Activity**
Protease inhibitors	Decrease activity of β- and γ-secretase that cleave Aβ from APP
Extracellular Aβ-binding compounds e.g. Cu^2+^, Zn^2+^ chelators	Prevent aggregation of Aβ into cytotoxic amyloid fibrils
Immunotherapeutic agents	Induces local and T-cell innate immune response.
Non-steroidal anti-inflammatory drugs, e.g. naproxen, celecoxib, aspirin	Dampens the innate immune-response thereby delaying the progression of AD
Neuroprotective agents e.g. antioxidants, MAO-inhibitors, Ca-channel blockers and anti-apoptotics	Interferes with the mechanisms of Aβ-triggered putative neurotoxicity
Statins, e.g. simvastatin	Decreases cholesterol which is a major risk factor in amyloid accumulation thereby lowering the risk of AD
Hormonal replacement, e.g. estrogens	Decreases the risk of developing AD in postmenopausal woman.
Cholinergic replacement agents and cholinesterase inhibitors, e.g. tacrine, donepezil, galantamine	Symptomatic treatment of AD
Trophic factors	Prevent degeneration of axotomized cholinergic septal and BF neurons by regulating hippocampal NGF, APP and ACh-mediated activity
Environmental enrichment agents	Lead to pronounced reductions in cerebral amyloid deposits thereby lowering the risk of AD

**Table 2.** Clinical symptoms of atypical and typical Parkinsonianism.

**Features**	**Atypical Parkinsonianism**	**Typical Parkinsonianism**
Pathological hallmark	Loss of substantia nigra cells and neuronal cell degeneration containing DA receptors in parts of the CNS such as striatum as well as Dementia with Lewy bodies (DLB) cortico-basal ganglionic degeneration (CBD)	Loss of substantia nigra cells, preserved cells in striatum (basal ganglia) and response to DA stimulation.
Clinical symptoms	Resting tremor, slowed movement, muscle rigidity, postural instability as well as vertical gaze palsy, early postural instability (progressive supranuclear palsy) and multiple system atrophy (anterocollis).	Resting tremor, slowed movement, muscular rigidity, postural instability.
Heredity and familyhistory	More sporadic than familial.	Sporadic and familial. Family history plays a major role.
Genetic involvement	Tau positive NFTs present as neuronal inclusions, no Lewy bodies.	Lewy bodies, mutation of α-synuclein, parkin and ubiquitin genes.
Aetiology	Sporadic, toxins, MPTP, viruses.	Mostly genetic, mitochondrial damage, cell protein disposal.
Inflammation	Inflammation of substantia nigra and basal ganglia.	Inflammation of the basal ganglia.
